# Tailored Magnetic Fe_3_O_4_-Based Core–Shell Nanoparticles Coated with TiO_2_ and SiO_2_ via Co-Precipitation: Structure–Property Correlation for Medical Imaging Applications

**DOI:** 10.3390/diagnostics15151912

**Published:** 2025-07-30

**Authors:** Elena Emanuela Herbei, Daniela Laura Buruiana, Alina Crina Muresan, Viorica Ghisman, Nicoleta Lucica Bogatu, Vasile Basliu, Claudiu-Ionut Vasile, Lucian Barbu-Tudoran

**Affiliations:** 1 Interdisciplinary Research Centre in the Field of Eco-Nano Technology and Advance Materials CC-ITI, Faculty of Engineering, “Dunărea de Jos” University of Galati, 47 Domnească, 800008 Galati, Romania; elena.herbei@ugal.ro (E.E.H.); alina.muresan@ugal.ro (A.C.M.); viorica.ghisman@ugal.ro (V.G.); nicoleta.simionescu@ugal.ro (N.L.B.); 2 Cross-Border Faculty, Cahul, “Dunărea de Jos” University of Galati, 47 Domnească Street, 800008 Galati, Romania; vasile.basliu@ugal.ro; 3 Clinical Medicine Department, Medicine and Pharmacy Faculty, “Dunarea de Jos” University of Galati, 47 Domneasca, 800008 Galati, Romania; ionut.vasile@ugal.ro; 4 Electron Microscopy Center “Prof. C. Craciun”, Faculty of Biology & Geology, “Babes-Bolyai” University, 5-7 Clinicilor St., 400006 Cluj-Napoca, Romania; lucian.barbu@itim-cj.ro; 5 Integrated Electron Microscopy Laboratory, National Institute for Research and Development of Isotopic and Molecular Technologies, 67-103 Donat St., 400293 Cluj-Napoca, Romania

**Keywords:** magnetic nanoparticles, Fe_3_O_4_ core–shell nanostructures, contrast agents, medical imaging, Vibrating Sample Magnetometry (VSM)

## Abstract

**Background/Objectives:** Magnetic nanoparticles, particularly iron oxide-based materials, such as magnetite (Fe_3_O_4_), have gained significant attention as contrast agents in medical imaging This study aimsto syntheze and characterize Fe_3_O_4_-based core–shell nanostructures, including Fe_3_O_4_@TiO_2_ and Fe_3_O_4_@SiO_2_, and to evaluate their potential as tunable contrast agents for diagnostic imaging. **Methods:** Fe_3_O_4_, Fe_3_O_4_@TiO_2_, and Fe_3_O_4_@SiO_2_ nanoparticles were synthesized via co-precipitation at varying temperatures from iron salt precursors. Fourier transform infrared spectroscopy (FTIR) was used to confirm the presence of Fe–O bonds, while X-ray diffraction (XRD) was employed to determine the crystalline phases and estimate average crystallite sizes. Morphological analysis and particle size distribution were assessed by scanning electron microscopy with energy-dispersive X-ray spectroscopy (SEM-EDX) and transmission electron microscopy (TEM). Magnetic properties were investigated using vibrating sample magnetometry (VSM). **Results:** FTIR spectra exhibited characteristic Fe–O vibrations at 543 cm^−1^ and 555 cm^−1^, indicating the formation of magnetite. XRD patterns confirmed a dominant cubic magnetite phase, with the presence of rutile TiO_2_ and stishovite SiO_2_ in the coated samples. The average crystallite sizes ranged from 24 to 95 nm. SEM and TEM analyses revealed particle sizes between 5 and 150 nm with well-defined core–shell morphologies. VSM measurements showed saturation magnetization (Ms) values ranging from 40 to 70 emu/g, depending on the synthesis temperature and shell composition. The highest Ms value was obtained for uncoated Fe_3_O_4_ synthesized at 94 °C. **Conclusions:** The synthesized Fe_3_O_4_-based core–shell nanomaterials exhibit desirable structural, morphological, and magnetic properties for use as contrast agents. Their tunable magnetic response and nanoscale dimensions make them promising candidates for advanced diagnostic imaging applications.

## 1. Introduction

Lately, nanotechnology applications in diagnostic medicine and different therapies have emerged as a promising field in terms of different nanoparticles named SPIONS, consisting of maghemite (γ-Fe_2_O_3_) and other metallic ferrites, especially superparamagnetic Fe_3_O_4_, used for drug delivery [1,2], imaging [2], tissue engineering and contrast agent for organs, tissues and cells [3], MRI (magnetic resonance imaging) of gastrointestinal tract, detect focal lesions in the liver and spleen and to separate metastatic and benign lymph nodes [4]. The uses of magnetic particles due to their versatility are also favorable in the treatment of different diseases, such as cancer therapy [5,6], cardiovascular disease and neurological disease [7]. The two key factors for successful biomedical application are water suspensibility and biocompatibility, in addition to the optimized superparamagnetic properties [8].

Advanced research in nanotechnology and materials science is leading to the proper use of magnetic nanostructures, and research into new magnetic effects is leading to the use of magnetic nanostructures, such as gadolinium (Gd) and manganese (Mn) based nanomaterials and iron oxide, as promising alternatives to commercial contrast agents. These contrast agents used in medical imaging for their high biocompatibility and the ease of metabolization and surface functionalization showed a saturation magnetization for magnetite varying from 33.4 to 51 emu/g, depending on the nanoparticles’ dimensions at low applied fields [9].

Because the conventional MR contrast agents based on gadolinium are not efficiently internalized by the targeted cells, iron oxide magnetic particles represent the most used contrast agents for cell identification and labelling. Chauveau et al. present three important categories of iron oxide that are used as MR agents: SPIO (superparamagnetic particles of iron oxide more than 50 nm), USPIO (ultrasmall SPIO smaller than 50 nm) and MPIO (micron-sized particles iron oxide with size bigger than 0.9 µm) [10].

Magnetite-Fe_3_O_4_ is a superparamagnetic material arranged in an inverse spinel structure, which is a double oxide of Fe_2_O_3_ and FeO with crystal-containing regions of unpaired spins [11]. In the absence of a magnetic field, the magnetic domains are in a disordered state, but once applied, they align to form a magnetic field that is bigger than the sum of all individual unpaired electrons. As M.E. Compeán-Jasso et al. present in their work, the nanoparticles have to be smaller than 20 nm in order to interact with cells and protein or tissue [12]. The particles’ dimensions depend on the synthesis method and on chemical factors, such as iron salts, temperature, pH, precipitation agents, ionic strengths and the use of agglomeration agents. Because of the physical interaction of the particles, new methods of magnetite synthesis are using different agents in order to stabilize the increasing particle dimensions and to ensure safety and low cytotoxicity in several cell lines, such as polyethylene glycol [13], poly (lactic-co-glycolic acid), polyvinyl alcohol poloxamers, poloxamines, oleic acid, and sodium citrate [14,15,16].

The synthesis and characterization of magnetite have been studied by many groups [9,15,16,17,18], and the main conclusion is that it is necessary to control the size and shape of particles in order to be used as a contrast agent. The main properties for magnetic resonance imaging depend on saturation magnetization [19] and the relaxation time of the water protons.

There are many methods of magnetite synthesis, including chemical, physical and biological. Here, we mention green route magnetite, hydrolysis, co-precipitation method, partial hydrolysis of ferrous hydroxide, hydrothermal or high-temperature reactions, sol–gel synthesis, wet reduction, thermal decomposition, sonochemical synthesis, gas-phase deposition, spattering, aerosol spray pyrolysis, electron beam deposition, and biological synthesis [17,19,20].

Other applications of magnetite are presented in the literature for antimicrobial properties [21], dopamine identification with screen-printed carbon electrode [22], smart drug-delivery systems, therapies against cancer cells, radiotherapy [15], magnetic hyperthermia applications [23].

This study presents the synthesis and characterization of magnetite at three different temperature syntheses, 94, 96 and 98 °C, and the influence of titania and silica on magnetite synthesis at 96 °C (Fe_3_O_4_@TiO_2_ and Fe_3_O_4_@SiO_2_) nanocomposites to be used for medical imaging as contrast agents.

The values of nanoparticle dimensions from 5 to 150 nm and magnetic saturation of the synthesized nanocomposites, varying from 40 emu/g to 70 emu/g, of the magnetite samples enables it to be used in medical imaging as a contrast agent. Usually, the uses of magnetic nanoparticles (MNP) for medical imaging are based on the modification of the nuclear magnetic resonance effect of water protons from tissue structure and the presence of magnetic particles that change the signal of water protons and MNP can be used as contrast agents for malignant tumor detection [20]. The aim of this study is to investigate how synthesis temperature influences the structural, morphological, and magnetic properties of Fe_3_O_4_-based nanoparticles, with and without TiO_2_ or SiO_2_ coatings. By conducting a comparative analysis, this work provides new insights into the tunability of particle size and magnetic response, critical parameters for optimizing their performance as contrast agents in medical imaging. The novelty of the study lies in the systematic correlation between synthesis conditions and magnetic behavior, highlighting the potential of core–shell Fe_3_O_4_ nanostructures for tailored diagnostic applications.

## 2. Materials and Methods

### 2.1. Synthesis of Fe_3_O_4_ Nanoparticles and Core–Shell Composites

All reagents used were of analytical grade and employed without further purification. The following chemicals were sourced from Sigma-Aldrich (St. Louis, MO, USA): ferric chloride hexahydrate (FeCl_3_·6H_2_O, ACS ≥ 99.0%), ferrous sulfate heptahydrate (FeSO_4_·7H_2_O, ACS ≥ 99.0%), sodium hydroxide (NaOH, ≥98%), TiO_2_ nanopowder (<100 nm, 99.5% trace metals basis), SiO_2_ nanopowder (5–20 nm, 99.5% trace metals basis), and distilled water.

#### 2.1.1. Synthesis of Fe_3_O_4_ Nanoparticles via Co-Precipitation

Magnetite nanoparticles were synthesized by co-precipitating Fe^3+^ and Fe^2+^ salts in a 2:1 molar ratio under alkaline conditions. Specifically, 0.2 mol/L FeCl_3_·6H_2_O and 0.1 mol/L FeSO_4_·7H_2_O were dissolved in 100 mL of distilled water and transferred to a three-neck round-bottom flask equipped with a thermometer, reflux condenser, and mechanical stirrer.

The solution was stirred vigorously (700 rpm) and heated to one of the target temperatures: 94 °C (P2), 96 °C (P1), or 98 °C (P5). After temperature stabilization, 0.1 mol/L NaOH solution was added dropwise until the pH reached 11–12. A black precipitate formed immediately, indicating Fe_3_O_4_ formation. The reaction was maintained for 2 h at constant temperature and stirring speed.

After synthesis, the precipitate was magnetically separated using an external magnet, washed thoroughly with distilled water (10 times) to remove unreacted salts and byproducts, and dried at room temperature in an oven for 7 days.

#### 2.1.2. Synthesis of TiO_2_- and SiO_2_-Coated Fe_3_O_4_ Nanoparticles

To obtain Fe_3_O_4_@TiO_2_ and Fe_3_O_4_@SiO_2_ core–shell nanoparticles, 0.1 wt% TiO_2_ or SiO_2_ (relative to Fe_3_O_4_ mass) was added to the iron salt solution prior to the NaOH addition. The pH was adjusted to 11–12 using NaOH, and the mixture was maintained at 96 °C with 700 rpm stirring for 2 h. The formation, separation, washing, and drying of the coated nanoparticles followed the same steps as for uncoated Fe_3_O_4_.

All syntheses were conducted in ambient air (no inert gas). Sample codes and synthesis conditions are summarized in Table 1.

For preparing various samples, the iron salts FeCl_3_∙6H_2_O-0, 2 mol/L and FeSO_4_∙7H_2_O-0.1 mol/ were mixed in a three-neck round bottom flask under vigorous stirring (700 rpm) at three temperature rates (sample particle named P1 for 96 °C, P2 for 94 °C and P5 at 98 °C) for about 2 h. After 2 h, the 0.1 mol/L NaOH was added to the yellow-orange mixture solution of iron salts, and solid black particles were formed. To prepare the nanocom-posites Fe_3_O_4_/TiO_2_ and Fe_3_O_4_/SiO_2_, 0.1 wt% (reported to the mass of magnetite) of TiO_2_ and SiO_2_ were added at the moment of alkaline precipitation in the mixture solution of iron salts at 96 °C. The sample particles named were P3 for 96 °C with TiO_2_ nps added and P4 for 96 °C with SiO_2_ NPs added. The synthesis took place in the air atmosphere. After the formation of black suspended particles, these were washed under vigorous agitation in distilled water 10 times to eliminate the unreacted chemicals and the secondary products. The particles were separated by an external magnet and then dried at room temperature in an oven for 7 days. After 7 days of resting, the solid particles were used for characterization (FTIR, XRD, SEM/EDX, TEM, VSM).

The synthesis temperatures (94 °C, 96 °C, 98 °C) were selected to explore thermal effects near the boiling point of water, where co-precipitation kinetics of Fe_3_O_4_ are highly sensitive to slight variations, as supported by prior studies [24,25].

Superparamagnetic nanoparticle magnetite synthesis is a complex process as described by Prabowo et al. [26]. The proposed reaction scheme for magnetite coprecipitation could be utilized to develop a more detailed study of the reaction mechanism. To obtain magnetite, additional steps are followed. The iron salts are decomposed in water and form ferrous and ferric hydroxide, and the trivalent hydroxide is decomposed to ferric oxy-hydroxide and then reacts with ferrous hydroxide and forms magnetite as presented below:

FeCl_3_(s) → Fe_3_^+^(aq) + 3Cl^−^(aq);

FeSO_4_.7H_2_O → Fe^2+^(aq) + SO_4_^2−^(aq) + 7H_2_O(l);

NaOH(s) + H_2_O(l) → Na^+^(aq) + OH^−^(aq);

Fe^3+^(aq) + 3OH^−^(aq) → Fe(OH)_3_;

Fe(OH)_3_(s) →FeO(OH) + 4H_2_O(l);

Fe^2+^(aq) + 2OH^−^(aq) → Fe(OH)_2_;

2FeO(OH) + Fe(OH)_2_ → Fe_3_O_4_(s) + 4H_2_O(l).

**Figure 1:**
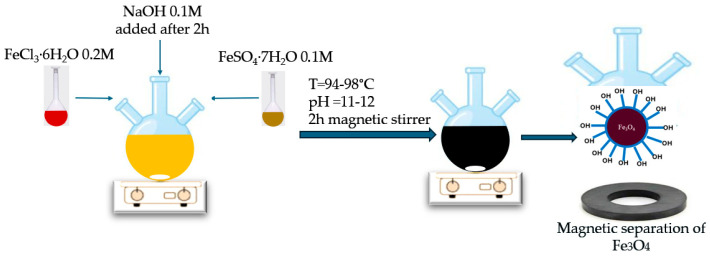
Schematic synthesis approach of magnetite by co-precipitation method.

The principal scheme of the magnetite synthesis via the co-precipitation method is represented in Figure 1.

The surface chemistry of magnetite particles is very important. The iron atoms on the surface of the magnetite particle that are not bound to oxygen atoms act as Lewis acids, and due to this property, they can donate a pair of electrons. In aqueous systems, these atoms coordinate the water molecules that dissociate rapidly, resulting in surface-functioning magnetite with hydroxyl groups of the Fe-OH type. For this reason, surface chemistry depends on the pH. At low pH, the surfaces are protonated, and at higher pH, the surface is negatively charged; that is why this magnetic particle can be functionalized with different agents or particles to modify the properties of materials. In this case, the hydroxyl groups that surround the magnetite core are amphoteric and can react with acid or base compounds. In our research for the Fe_3_O_4_/TiO_2_ and Fe_3_O_4_/SiO_2_, by adding titania and silica at the moment of solution precipitation, oxides surround the magnetite core due to the physical interaction determined by the amphoteric character of hydroxyl groups. To maintain monodisperse nanoparticles, it is necessary to control the nucleation. The control of nucleation depends on factors such as pH adjusting, ionic strength, the nature of salt, temperature, molar ratio of Fe^3+^/Fe^2+^, and the concentration of the precipitation agent [27]. The pH can significantly affect the solubility of the precursors used in synthesis. For instance, in the case of metal oxide nanoparticles, a higher pH might lead to the precipitation of metal hydroxides, which then serve as nuclei for particle growth. In our synthesis, we adjust pe pH in the range of 11–12 to have a constant growth rate and to decrease the trend of agglomeration.

### 2.2. Characterization Methods

The IR spectra of the samples were measured within the range of 4000–400 cm^−1^ by the FTIR-attenuated total reflection (FTIR-ATR) method, which records spectra through attenuated total reflection. The P1 to P5 spectra were recorded with a resolution of 2 cm^−1^, and each scan was repeated 45 times. The FTIR spectrometer was operated within a temperature-controlled (20 °C) air-conditioned chamber. The IR spectra of solid magnetite were captured using an IR-Spirit-T FTIR Spectrometer from Shimadzu Corporation, headquartered in Kyoto, Japan, equipped with a built-in ATR accessory type QATR-S, DLATGS detector, and KBr beam splitter.

For the confirmation of crystallinity and phase formation of the P1–P5 samples X-ray diffraction was performed on Dron-3 equipment with CoK radiations (λ = 1.790300 Å) X-ray diffractometer operated at a voltage of 30 kV and 20 mA currents, with a step of 0.05 °/s, a time exposure of 3 s, total time/sample 2 h and 13 min, in a range of 2θ = 15–900. The diffracton patterns obtained were analyzed using the software Match! 4 (using Rietveld refinement), and the reference database library used is the Crystallography Open Database (COD). The average crystallite of magnetite was calculated using Scherrer’s equation for the most intense peak (311); D = kλ/(β·cosθ) (1), where k = 0.94, λ is the wavelength of the X-ray (λ = 1.7902 Å), β is full width at the half maximum (FWHM) of the peak, which is the diffraction angle.

The structure, elemental composition, and morphology of the developed nanomaterials were examined by scanning electron microscopy in high vacuum conditions with a 4th-generation TESCAN VEGA electron source and tungsten filament, which combines SEM imaging and elemental composition analysis directly in a single Essence™ from TESCAN software (1.2.1.0 build 5762, March 2023) window. The powder samples were analyzed as obtained after the drying process.

The morphology of the magnetic nanoparticles in the colloidal suspension was observed with a scanning transmission electron microscope STEM (Hitachi, Tokyo, Japan). A drop of 7 µL of sample was deposited onto 400 mesh Cu grids and examined with a Hitachi HD-2700 CFEG STEM (Hitachi, Tokyo, Japan) and windowless double EDX detectors X-Max 100 (Oxford Instruments, Abingdon, UK).

The magnetic properties of the produced iron oxide nanomaterials were evaluated at room temperature by using a vibrating sample magnetometer (VSM), the LakeShore model 8607 (Lake Shore Cryotronics, Westerville, OH, USA). Magnetic measurements were recorded under an applied magnetic field of 30 kOe. Before each experiment, the investigated material was demagnetized in alternating fields. The working temperature of the samples analyzed was around 230 °C. The iron oxides were used as they were prepared without any other sample preparation.

Ethical Statement: This study did not involve human participants, animal subjects, or any procedures requiring institutional approval. Therefore, Institutional Review Board (IRB) or Ethics Committee approval was not required.

## 3. Results

### 3.1. Structural Characterization of Magnetite Nanomaterials

**Figure 2:**
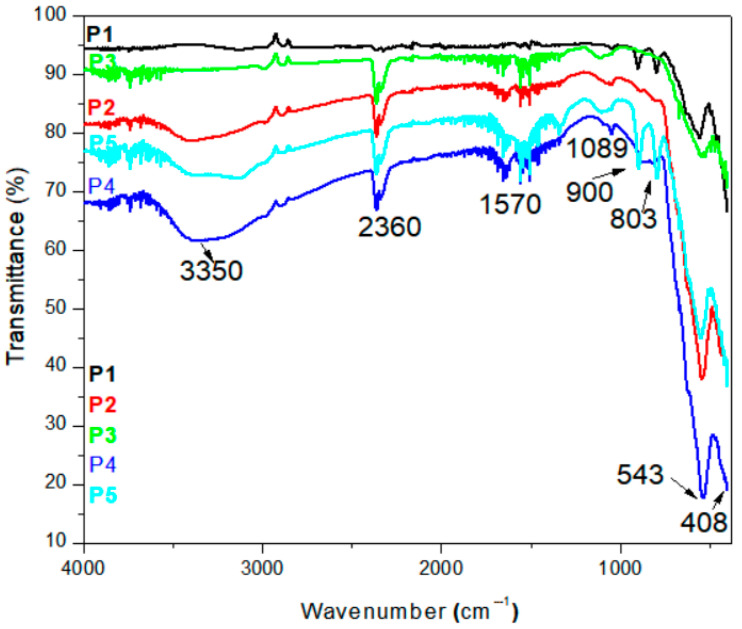
Comparison of FTIR spectra for magnetic nanomaterials (P1 to P5).

The resulting FTIR spectra of iron oxide nanomaterials obtained by coprecipitation synthesis used in this study are presented in Figure 2. The spectra presented are compared for all the samples. It can be observed that we have the common large band centered at 3350 cm^−1^ (O–H stretching vibrations) and 2360 cm^−1^ O–H bending vibrations [28,29]. The band at 1570 cm^−1^ is due to water molecules adsorbed on the magnetite surface [30].

**Figure 3:**
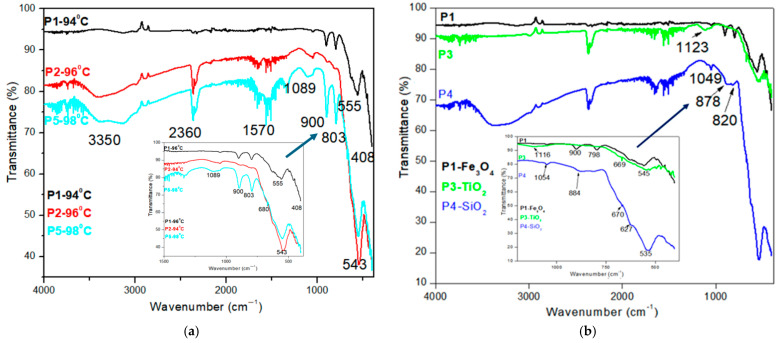
(**a**) Comparison of FTIR spectra for magnetic iron oxide nanomaterials at different temperatures and coprecipitation conditions. (**b**) Comparison of FTIR spectra for Fe_3_O_4_, Fe_3_O_4_@TiO_2_ and Fe_3_O_4_@SiO_2_.

Regarding metal oxides bonds for iron oxides, titania, and silica, peaks are illustrated in Figure 3a,b. In Figure 3a, we compared the samples by temperature influences. For the three temperatures, we observe the bands from 543 to 555 cm^−1^ common to all temperatures. This band is assumed to be the Fe-O stretch from the magnetite compound [29,31]. The vibrational bands that appear between 803 and 900 cm^−1^ can be attributed to the FeOOH [32]. The bands were attributed to the Fe–O vibrational modes in β-FeOOH (transformation phase from FeOOH to Fe_3_O_4_ from the synthesis) [2]. For each sample, the presence of Fe-O from the magnetic iron is demonstrated. The temperature of synthesis influences the order of peak intensity. The bands from 803 to 900 are very low at 94 °C and increase at 96 °C and 98 °C.

Figure 3b illustrates the FTIR spectra of coated magnetite Fe_3_O_4_, Fe_3_O_4_@TiO_2_, and Fe_3_O_4_@SiO_2_, respectively. For the sample with TiO_2_ nanoparticles, we have an absorption band around 669 and 1123 cm^−1^ [32]. The characteristic peaks for silica-coated magnetite were observed at 820, 879 and 1049 cm^−1^ owing to Si-O-Si symmetric stretching, Si-OH stretching, and Si-O-Si asymmetric stretching vibrations, respectively [33,34]. In the FTIR spectra (Figure 3b), the absorption bands attributed to Ti–O and Si–O bonds are present but relatively weak, which is consistent with the low coating concentration (0.1 wt%) and partial surface coverage. The weak intensities of the bands at 820, 879, and 1049 cm^−1^, characteristic of silica, suggest the presence of SiO_2_ in trace amounts or as a discontinuous surface coating. These peaks should be interpreted cautiously, as they may also overlap with vibrational modes from iron oxides or adsorbed species.

Because of its low concentration (seen also in EDX spectra), the peaks for SiO_2_ nanoparticles are very low in intensity. The FTIR spectra reveals the presence of iron oxides, silica, and titania particles used to coat the iron oxide and to observe the influence on magnetic properties.

**Figure 4:**
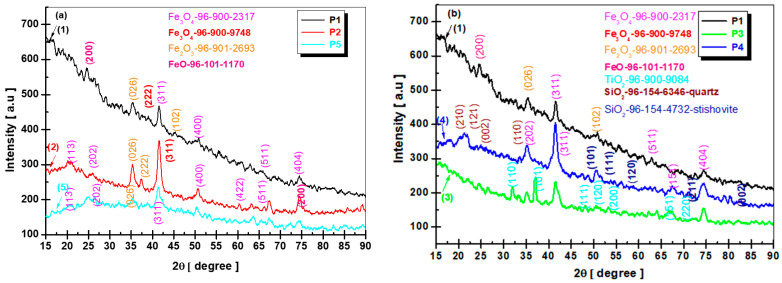
(**a**) XRD patterns for magnetite synthesized at 94, 96 and 98 °C. (**b**) XRD patterns for Fe_3_O_4_, Fe_3_O_4_/TiO_2_ and Fe_3_O_4_/SiO_2_.

The X-ray diffractograms of all the samples synthesized at various temperatures are shown in Figure 4a,b. The crystalline phase was identified using the Crystallography Open Database (COD). The particles synthesized at pH interval 11–12 at temperatures from 94 to 98 °C were identified as owing to the cubic magnetite as reported in the literature [35,36,37,38,39] for a pattern with diffraction peaks at 2θ of 20°, 25°, 45.5°, 50°, 51.5°, 74° and 75° relative to the diffractions lattice of (113), (202), (311), (400), (511), (200) and (404) crystal faces of the Fe_3_O_4_ spinel structure (COD-96-900-2317). For all three temperatures, we find peaks corresponding to Fe_2_O_3_: (026), (202), and (102) crystal phases (COD-96-901-2963) and Fe-O (200) crystal phase (COD-96-101-1170). Regarding the temperature for the P2 sample, a better crystallization of peaks was observed.

Figure 4b presents the phase identification and crystalline structure of the as-prepared black MNPs for the 96 °C of magnetite, titania, and silica-coated magnetite. As shown in Figure 4b, the diffraction peaks of the sample could be readily assigned to the cubic phase Fe_3_O_4_ following the main characteristic peaks for magnetite presented in Figure 4a. The diffraction peaks obtained imply also the characteristic peaks for TiO_2_-rutile tetragonal phase (curve 3-green) with diffraction peaks at (110), (101), (111), (120), (211), and (220) (COD-96-900-9084). The sample with magnetite silica coated reveals the tetragonal stishovite phase identified using (COD-96-900-7151). Generally, diffraction peaks were obtained for SiO_2_ at (110), (111), (120), (211) and (002), respectively [34]. In Figure 4b, the diffraction peaks assigned to TiO_2_ (rutile) and SiO_2_ (stishovite) are observed with low intensity, reflecting either partial crystallinity or a trace-level presence of the coatings. Given the low weight fraction used (0.1%), the detection of TiO_2_ and SiO_2_ phases via XRD may be limited by signal sensitivity and peak overlap with dominant Fe_3_O_4_ reflections. Therefore, the identification of these phases should be considered semi-quantitative.

From the diffraction patterns, using the software Match! 4 (using Rietveld refinement), the reference database library used is the Crystallography Open Database (COD). Fe_3_O_4_’s lattice parameters and crystallite size are listed in Table 2. XRD patterns were calculated for the average crystallite size. Comparing the crystallite average of the sample, we can observe that for 94 °C it is 24 nm, for 96 °C it is 27 nm and at 98 °C it is 95 nm, an increasing value as the temperature increasing. Adding TiO_2_ for coating magnetite determines the increase of nanoparticles to 64 nm. Because silica is very low in percentage, the increase in magnetic nanoparticles is proportional to the low rate (0.2%) of covered silica.

The highest intensity of the diffraction peak of magnetite (311) was analyzed using the Scherer equation, generating particle sizes of from 20 to 150 nm for the samples presented. Comparing the literature data, we obtained a larger crystallite size of magnetite, but we did not use any functionalizing agent to maintain a constant nucleation rate.

### 3.2. Structural Analysis

**Figure 5:**
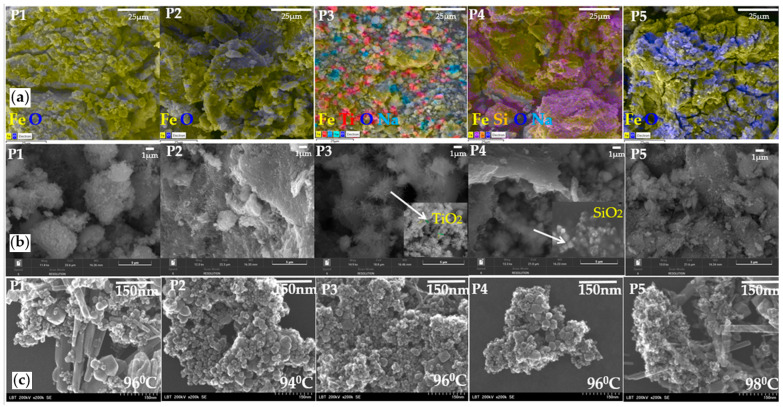
SEM images of magnetic nanoparticles (**a**) elemental analysis (25 µm), (**b**) top view (1 µm) and (**c**) top view (150 nm).

Figure 5 presents the SEM morphology for the studied nanocomposites’ surfaces. The elemental composition (EDS) presents a uniform arrangement of magnetite constituents. (Figure 5a) The top view (1 µm) of magnetite samples generally indicates the agglomeration of magnetite and also the coated magnetite with titania and silica. The mapping elements show a good distribution of titania onto magnetite particles (Figure 5a—P3) and a weaker particle distribution for SiO_2_ (Figure 5a—P4). The images at a 25 µm scale show the same type of agglomeration of magnetite particles. Only for the sample with titania is a decrease in agglomeration observed. Regarding the temperature variation, no difference was observed in morphology. The agglomeration of particles is almost the same for each structure. Figure 5b indicates the magnetite agglomeration (Figure 5b—P1, P2, P5) and the presence of TiO_2_ nanoflowers and SiO_2_ nanoflakes among magnetite particles (Figure 5b—P3) and (Figure 5b—P4), respectively. White arrows indicate the presence of TiO_2_ and SiO_2_ nanostructures in the coated Fe_3_O_4_ nanoparticles (P3 and P4, respectively). The silica is observed covering the magnetite as an agglomerated snowflake about 20 nm in dimension (Figure 5b—P4). Figure 5c shows the top-view images of the nanocomposites studied at 150 nm. Particulate irregularly agglomerated morphologies with a high distribution of particles, with the average size around 20 nm for nanospheres and 150 nm for magnetite nano baguettes, were observed (Figure 5c). SEM images also revealed different morphologies such as nanospheres, rods and flowers from 15 nm to 150 nm.

Although SEM images (Figure 5a) suggest that P_4_ (SiO_2_-coated) forms larger visible agglomerates, the XRD-derived crystallite size is smaller than that of P_3_. This may be due to partial amorphization or the porous structure of silica, which leads to low-intensity diffraction peaks and a smaller calculated crystallite size.

**Figure 6:**
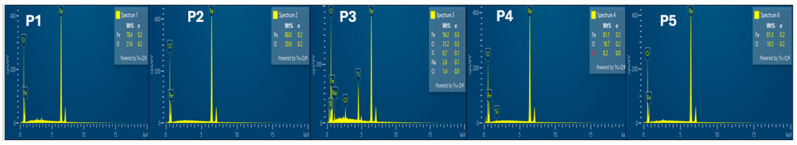
EDX elemental analysis of the resulting magnetite P1-96 °C, P2-94 °C, P5-98 °C and magnetite nanocomposites P3-Fe_3_O_4_@TiO_2_ nanoparticles, P4 Fe_3_O_4_@SiO_2_ nanoparticles.

The EDX analysis of the Fe_3_O_4_ nanoparticles and the list of its composition were given in Figure 6, which proves the presence of iron and oxygen without any sign of impurity (the presence of Na ions is observed only for the samples with TiO_2_, probably due to the precipitation agent). The percentage of Fe varies from 78 to 81% and oxygen from 19 to 22%, as observed in Figure 6.

**Figure 7:**
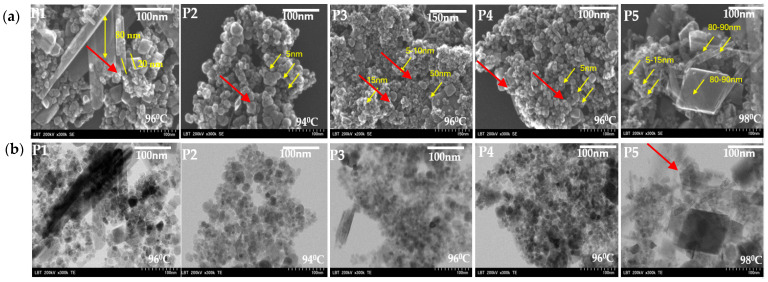
A general view of P1–P5 magnetite nanomaterials. (**a**) SEM top view of nanoparticles agglomeration of spherical, cubic and rod-shaped nanoparticles (**b**) TEM, a snapshot of an agglomerate of amorphous particles of Fe_3_O_4_ NPs.

Figure 7 shows the SEM (Figure 7a) and TEM (Figure 7b) micrographs of Fe_3_O_4_ nanoparticles. The particles are almost spherical and cubic in the same areas, with diameters ranging from 5 nm for spherical nanoparticles to 80–90 nm side for cubic nanoparticles. We can observe, for samples P1 and P5, the presence of cubic particles sprinkled for 96 and 98 °C. Generally, all the samples present highly distinguishable interparticle mesopores. For the samples with titania and silica, we can see quasi-spherical nanoparticles in the majority. These particles are polydisperse and some of them agglomerated, due to magneto–dipole interactions between particles. The temperature variation does not show a clear change in the size of the nanoparticles. Usually, the temperature rise should cause a decrease in the size of the nanoparticles. Elevated temperatures can enhance reaction rates, resulting in smaller particles due to rapid nucleation.

The most interesting component of the NP is a spatial 3D agglomerate up to 300 nm in size (shown in the circle on Figure 7a) consisting of nanoparticle aggregates and pores (a separate nanoparticle aggregate of about 100 nm in size is shown with the red arrow on Figure 3b). The SEM and TEM images (Figure 5 and Figure 7) reveal significant aggregation of Fe_3_O_4_ nanoparticles across all samples, particularly for uncoated magnetite. This aggregation can be attributed primarily to magnetic dipole–dipole interactions between nanoparticles, which is a well-known phenomenon in superparamagnetic systems. Additionally, van der Waals forces and the absence of strong surface functionalization contribute to particle clustering. During synthesis, the pH range of 11–12 may have led to partial surface charge neutralization, reducing electrostatic repulsion and promoting aggregation. Notably, in the TiO_2_-coated sample (P3), a reduction in agglomeration was observed, likely due to the physical barrier provided by the coating that weakens magnetic interactions. In contrast, the SiO_2_-coated particles (P4) exhibited a “snowflake-like” morphology, indicating partial surface coverage and persistent interparticle forces. Furthermore, some degree of aggregation may have occurred during sample drying for electron microscopy, which is typical in high-surface-area nanopowders.

### 3.3. Magnetic Properties

**Figure 8:**
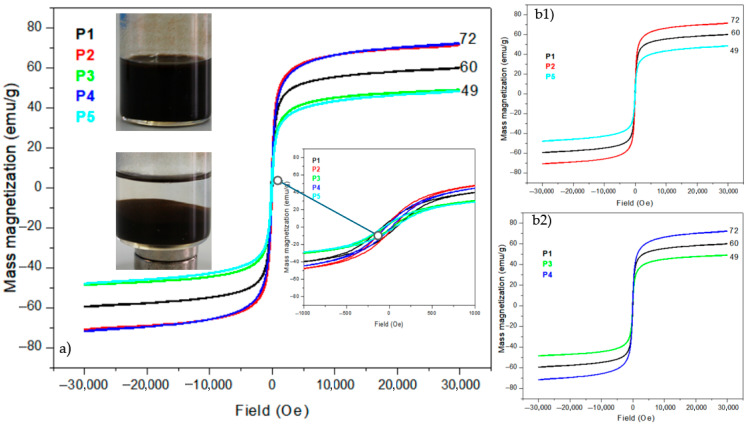
(**a**) Vibrating sample magnetometers curve of Fe_3_O_4_, Fe_3_O_4_/TiO_2_ and Fe_3_O_4_/SiO_2_ at different temperatures, (**b1**) Comparison of magnetization for 94, 96, and 98 °C, (**b2**) comparison of magnetization curves for Fe_3_O_4_, Fe_3_O_4_/TiO_2_ and Fe_3_O_4_/SiO_2_.

The magnetic properties of the obtained nanomaterials (Fe_3_O_4_, Fe_3_O_4_@TiO_2_, and Fe_3_O_4_@SiO_2_) were investigated using the vibrating sample magnetometer at a temperature of 296 K. The loops of the magnetic hysteresis (illustrated in Figure 8) suggested that the prepared composites showed ferrimagnetic behaviors. The inset image of Figure 8a presents the external magnetic separation of magnetic materials from the solution. In Figure 8a, we compared the five samples, and the largest value is 72 emu/g for the samples at 94 °C for Fe_3_O_4_ and at 96 °C for silica-coated magnetite. The lowest value of magnetization is 49 emu/g and is attributed to the samples at 98 °C and titania-coated magnetite at 96 °C. From the curve of the graph, it is confirmed that the synthesized Fe_3_O_4_ nanoparticles possessed nearly superparamagnetic behavior. In our work, we have lower values of saturation magnetization compared to the theoretical value of bulk magnetite (92 emu/g) [40,41].

**Figure 9:**
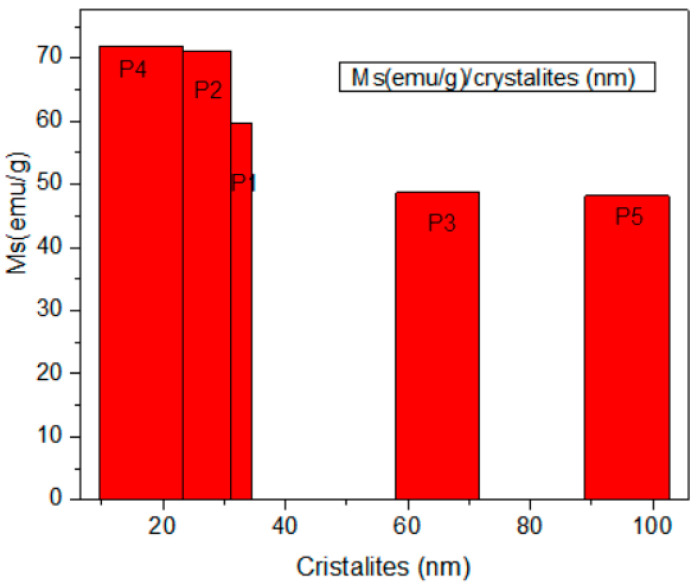
Comparison of Ms (emu/g) with magnetite crystallites (nm).

This can be explained as the introduction of non-magnetic materials (for the case of titania and silica nanoparticles) or because of the presence of lower magnetic materials, such as Fe_2_O_3_ iron oxides (72 emu/g), on the synthesized compound, leading to the diminution of magnetization. Also, the reduced particle sizes can cause a decrease in magnetite magnetization. These magnetic parameters are significant because the values are big enough to be used in medical imaging as a contrast agent. From the data in the literature, the values of magnetization depend on the method and the dimension of nanoparticles [20,30,42,43,44,45,46,47]. Figure 9 presents a comparison of magnetization values versus magnetite crystallite dimensions for our synthesis.

In our synthesis, the samples P4 and P2 with 16 to 24 nm cristalites on average showed the largest value for Ms of around 70 emu/g, as presented in Figure 9. The particle size and surface coating play a significant role in the high field irreversibility, high saturation field, and superparamagnetism of magnetic NPs. While a general trend can be observed between crystallite size and saturation magnetization, this is not strictly monotonic across all samples. For example, sample P_5_, despite its larger crystallite size (~95 nm), showed lower magnetization (48 emu/g), possibly due to increased multi-domain behavior or surface oxidation. Therefore, size must be considered alongside morphology and surface chemistry.

**Figure 10:**
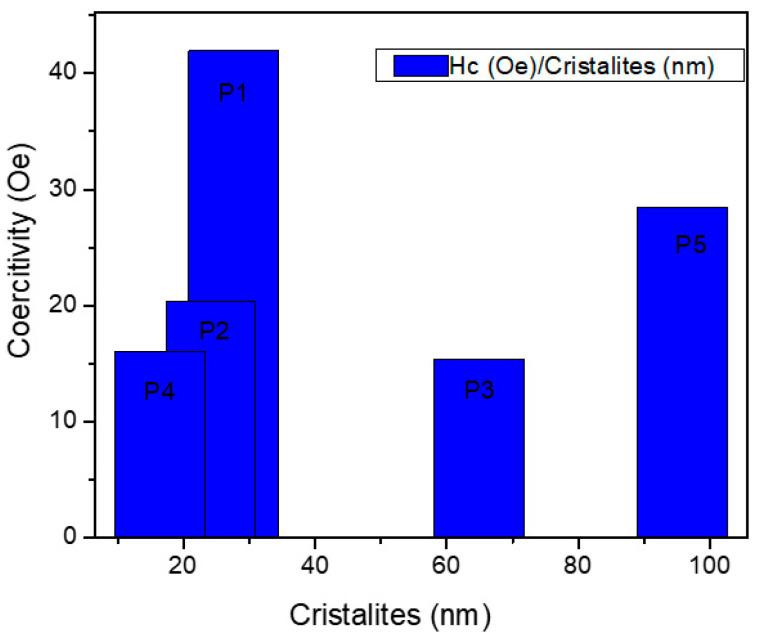
Comparison of Hc (Oe) with magnetite crystallites (nm).

Figure 10 shows the coercivity of magnetite nanoparticles. The largest value of Hc is for the sample obtained at 96 °C and 27 nm cristalite average. Bulk nanoparticles have a gradual increase in coercivity, which reaches a maximum value at a particular size. According to theoretical calculations, cubic and spherical magnetite nanoparticles become multi-domain when their critical size exceeds 76 nm and 128 nm, respectively [48]. Table 3 presents the main magnetization properties extracted from vibrating sample magnetometer measurements.

In accordance with the data in the literature, by decreasing the size of nanoparticles, coercivity rapidly drops to zero, reaching a superparamagnetic state [49,50]. In our case, the lowest value for Hc is assumed to be for Fe_3_O_4_@TiO_2_. Superparamagnetism is needed in applications such as drug delivery and imaging. It requires particle sizes below 20 nm, as shown in Table 4, which presents a comparison of magnetic properties with the synthesis method and nanoparticle dimension.

A broader comparison with the literature shows that magnetite nanoparticles synthesized by the co-precipitation method typically exhibit saturation magnetization (Ms) values ranging from 50 to 90 emu/g, depending on synthesis conditions, particle size, and surface modification. For instance, Qi et al. [30] reported Ms ≈ 92 emu/g for 10 nm spherical Fe_3_O_4_ synthesized at 100 °C without any coating. Liu et al. [43] achieved Ms = 85 emu/g using hydrothermal synthesis for 40 nm cubic particles. In contrast, Ahn et al. [45] obtained Ms values around 70 emu/g for 15 nm magnetite particles designed for hyperthermia applications. Compared to these, our highest Ms value (~72 emu/g) for uncoated Fe_3_O_4_ at 94 °C (P2) falls within the expected range, particularly considering the synthesis method and particle size (24 nm). For coated samples, our Ms values dropped to 48–60 emu/g due to the non-magnetic TiO_2_ and SiO_2_ shells, in agreement with previous findings [20,41,47]. The coercivity and remanence values are similarly consistent with partially superparamagnetic behavior reported for Fe_3_O_4_ nanoparticles larger than 20 nm [46,50]. These comparisons reinforce that the magnetic behavior of our nanomaterials is within standard expectations, while the synthesis method offers the advantage of size and morphology control without toxic reagents.

## 4. Discussion

The synthesized Fe_3_O_4_-based nanoparticles demonstrated characteristic features consistent with prior studies using the co-precipitation method. The FTIR spectra revealed broad bands around 3350 cm^−1^ and 2360 cm^−1^ due to O–H vibrations, indicating surface hydroxyl groups [28,29], which are commonly reported for magnetite nanoparticles stabilized in aqueous environments. The presence of Fe–O vibrations near 543–555 cm^−1^ aligns with typical assignments in literature for magnetite nanostructures [29,31]. Additional bands between 803 and 900 cm^−1^ are attributed to FeOOH intermediates, confirming partial transformation pathways from Fe(OH)_3_ to Fe_3_O_4_ [2].

When TiO_2_ and SiO_2_ were introduced, new bands around 669, 820, and 1049 cm^−1^ appeared, which agree with Ti–O and Si–O–Si/Si–OH stretching vibrations previously reported for core–shell Fe_3_O_4_ systems [32,33,34]. Compared to studies by S. Hu et al. [42], our FTIR bands confirm effective surface functionalization, despite the low loading percentages.

XRD analysis confirmed the formation of the cubic spinel structure of Fe_3_O_4_ (COD 96-900-2317), consistent with findings by Mahdavi et al. [36] and others [36,37,38,39]. Coating with TiO_2_ and SiO_2_ slightly modified peak intensities, suggesting partial coverage and possibly strain at the interface. TiO_2_ was identified in the rutile phase (COD-96-900-9084), while silica appeared in the stishovite phase (COD-96-900-7151), in agreement with coatings described by Tadic et al. [33].

The average crystallite size increased with synthesis temperature, from 24 nm at 94 °C to 95 nm at 98 °C, which supports the trend reported by R. Massart [40], where higher synthesis temperatures lead to larger magnetite crystallites due to faster growth kinetics.

The selection of temperatures at 94 °C, 96 °C, and 98 °C was intentional to evaluate the effect of subtle thermal variations near the boiling point of water, a critical range for controlling nucleation and growth in co-precipitation reactions. This temperature interval is supported by literature reporting optimal Fe_3_O_4_ nanoparticle synthesis in the 90–100 °C range to ensure proper hydrolysis and phase formation (Refs: [30,40,43]). The results indicate that temperature had a significant influence on crystallite size and magnetic properties. Specifically, a progressive increase in temperature led to larger crystallite sizes (from 24 nm at 94 °C to 95 nm at 98 °C) and a decrease in saturation magnetization (from 72 to 48 emu/g). Morphological analysis showed minimal change in particle shape but a slight increase in agglomeration at higher temperatures. Therefore, temperature serves as a critical parameter in tuning both the physical and magnetic characteristics of Fe_3_O_4_-based nanoparticles.

The magnetic hysteresis loops of the coated samples (P3, P4) exhibited reduced coercivity (Hc) and remanent magnetization (Mr) compared to uncoated Fe_3_O_4_, which suggests a partial suppression of magnetic interactions due to the non-magnetic shell. However, the magnetic parameters do not fully satisfy the criteria for ideal superparamagnetic behavior, as the hysteresis loops still display non-zero Hc and Mr values. Notably, P3 (TiO_2_-coated) showed an Hc of 15.39 Oe and Mr of 2.34 emu/g, indicating a soft ferromagnetic response rather than true superparamagnetism. These findings suggest that while the coatings influence the magnetic softness of the nanomaterials, further size reduction and surface functionalization would be necessary to achieve superparamagnetism as defined by standard criteria [48,49,50].

SEM and TEM analyses confirmed the presence of mixed morphologies: nanospheres, rods, and cubic structures in the 5–150 nm range. Compared to the study of Tartaj et al. [43], our particles exhibited similar polydispersity but better uniformity for TiO_2_-coated samples. Silica coating led to flake-like morphology, similar to the findings of Lee et al. [45].

The magnetic measurements confirmed ferromagnetic behavior with saturation magnetization (Ms) ranging from 48 to 72 emu/g. While slightly lower than bulk Fe_3_O_4_ (92 emu/g) [40], these values fall within expected ranges for coated nanoparticles and align with other co-precipitation studies [20,30,43,44,45,46,47]. For example, our silica-coated sample (P4) showed 71.84 emu/g, comparable to SiO_2_-coated magnetite (70 emu/g) in Z. Yang et al. [44]. The reduction in Ms after coating is attributed to the presence of non-magnetic shells, consistent with previous work [30,48].

Coercivity (Hc) values were lowest (15.39–41.95 Oe) for samples with crystallite sizes below 30 nm, confirming the onset of superparamagnetism—a property desirable in imaging and drug delivery applications. This behavior was also reported by Kodama et al. [49] and Deatsch et al. [50], reinforcing the biomedical applicability of our synthesized materials.

The SiO_2_-coated sample (P_4_) exhibited high saturation magnetization (~71.8 emu/g), which is comparable to the uncoated sample P_2_ (~72 emu/g). This may result from its small crystallite size (16 nm), which is close to the superparamagnetic limit. The coating may have partially suppressed surface spin disorder, but the overall increase in magnetization compared to other samples is not significant enough to be attributed to the coating alone.

In summary, our findings confirm the successful synthesis of core–shell Fe_3_O_4_@TiO_2_ and Fe_3_O_4_@SiO_2_ nanostructures with properties comparable to or improved over previous reports. Compared to literature values (Table 4), our nanoparticles demonstrate competitive magnetic behavior, and their size range is within optimal limits for medical imaging applications.

While the synthesized Fe_3_O_4_-based nanocomposites coated with TiO_2_ and SiO_2_ demonstrate promising magnetic properties suitable for imaging applications, the toxicity profile of these materials was not assessed in the current study. Although Fe_3_O_4_, TiO_2_, and SiO_2_ are individually known for their relatively low toxicity and FDA acceptance in certain biomedical contexts, the combined core–shell structure, particle size, and surface chemistry can significantly influence cytotoxicity and biocompatibility. Furthermore, the magnetic properties alone are not sufficient to determine their suitability for in vivo applications. Therefore, future studies will focus on detailed in vitro cytotoxicity assays, hemolysis testing, and cellular uptake studies, followed by in vivo safety evaluations to fully establish their potential as safe contrast agents.

One limitation of this study is the absence of Mössbauer spectroscopy, which would provide direct information about the oxidation state of iron and help distinguish between magnetite (Fe_3_O_4_) and maghemite (γ-Fe_2_O_3_). Future work should include this technique for a more comprehensive analysis of the iron oxide phases.

## 5. Conclusions

Fe_3_O_4_ nanoparticles were successfully synthesized via coprecipitation at 94, 96, and 98 °C, and core–shell structures coated with TiO_2_ and SiO_2_ were obtained at 96 °C. FTIR analysis confirmed the presence of characteristic Fe–O, Ti–O, and Si–O bonds, while XRD revealed the formation of crystalline magnetite, with crystallite sizes ranging from 16 to 95 nm. SEM-EDX and TEM imaging showed spherical and cubic Fe_3_O_4_ particles (5–90 nm), with well-distributed nanoscale TiO_2_ and SiO_2_ coatings. Coating led to morphological changes, including the disappearance of cubic structures and the formation of flower-like and spherical agglomerates in the silica and titania samples.

Magnetic measurements indicated that uncoated Fe_3_O_4_ nanoparticles synthesized at 94–96 °C exhibited the highest saturation magnetization (~72 emu/g). The presence of TiO_2_ and SiO_2_ coatings reduced the magnetization to ~60 emu/g and ~40 emu/g, respectively, due to the non-magnetic shell and structural modifications. The lowest coercivity was observed in titania-coated samples, suggesting improved superparamagnetic behavior.

These findings highlight that magnetite nanoparticles synthesized via a simple coprecipitation route can exhibit favorable magnetic properties for use as contrast agents in medical imaging. Future work will focus on biopolymer surface functionalization to improve colloidal stability, reduce cytotoxicity, and enhance biomedical compatibility.

Limitations of this study include the absence of in vitro or in vivo biological assessments, such as cytotoxicity, hemocompatibility, or MRI imaging efficiency in biological media. Additionally, surface stability in physiological conditions and long-term dispersion behavior were not evaluated. The study also relied on fixed concentrations of coating agents without exploring their optimization.

Future work will focus on biopolymer-based surface functionalization (e.g., PEG, dextran, chitosan) to improve colloidal stability, reduce cytotoxicity, and enhance biocompatibility. Further, in vitro and in vivo evaluations will be necessary to confirm the efficacy of these core–shell nanoparticles as MRI contrast agents. Studies on targeted functionalization for specific imaging or therapeutic applications (e.g., tumor targeting, drug delivery) are also recommended.

## Figures and Tables

**Table 1:** Solid sample name and condition of synthesis.

Sample Name	Composition	Temperature Synthesis (°C)	Time	Final pH Solution
P1	Fe_3_O_4_	96	2 h/700 rpm	11–12
P2	Fe_3_O_4_	94	2 h/700 rpm	11–12
P3	Fe_3_O_4_/TiO_2_ (0.1 wt%)	96	2 h/700 rpm	11–12
P4	Fe_3_O_4_/SiO_2_ (0.1 wt%)	96	2 h/700 rpm	11–12
P5	Fe_3_O_4_	98	2 h/700 rpm	11–12

**Table 2:** Composition and structural parameters of investigated MNPs.

Sample Code	T (°C)	Elemental Percent from EDX Analysis (%)				Corelated Phases Composition from XRD	2θ (311)	FWHM (°)	Lattice Parameter (Å)	Crystalite Size (DS) (nm)
		Fe	O	Si	Ti					
P1	T = 96	78.4	21.6	-	-	Fe_3_O_4_	34.89	0.35	8.39650	27.64
P2	T = 94	80	20	-	-	Fe_3_O_4_	34.67	0.4	8.39850	24.17
P3	T = 96	56.2	31.2	8.7	-	Fe_3_O_4_, Fe_2_O_3_, FeO, TiO_2_-rutile	37.06	0.15	8.39650	64.90
P4	T = 96	81.8	18.7	-	0.2	Fe_3_O_4_ Fe_2_O_3_, FeO, SiO_2_-stishovite	41.49	0.6	8.3941	16.42
P5	T = 98	81.5	18.5	-	-	Fe_3_O_4_	31.22	0.1	8.39650	95.83

**Table 3:** Cristalites average for P1 to P5, Hc, Ms and Mr of different Fe_3_O_4_ nanoparticles.

Sample	Crystalite Size (DS) (nm)	Coercivity (Hc) Oe	Intrinsic Coercivity (Hci) Oe	Remanent Mass Magnetization (Mr) emu/g:	Saturation Mass Magnetization (Ms) emu/g
P1	27.64	41.95	81.20	6.83	59.68
P2	24.17	20.39	35.32	3.86	71.07
P3	64.90	15.39	32.49	2.34	48.68
P4	16.42	16.07	28.64	2.91	71.84
P5	95.83	28.44	62.91	4.11	48.14

**Table 4:** Comparison of magnetic properties with synthesis method and nanoparticle dimensions.

Methods	MNP Size and Shape (nm)	Ms (emu/g)	Hc (Oe)	T (°C)	Application	Ref.
Hydrothermal	40 Cubic	85	Hc-no data available	80 °C	Magnetic resonance imaging	[43]
Chemical synthesis	5–19 spheres, cubes, rods, and other shapes	54–101	Hc-no data available		Bioimaging	[42]
Co-precipitation	6 cubic	54	8–13	75 °C	Catalyst	[40]
Co-precipitation	11 all particles with a nearly spherical shape	60	Hc-no data available	75 °C	Coating agents	[41]
Co-precipitation	10 Crystalline, sphere	92	Hc-no data available	100 °C	Anti-cancer therapeutic and magnetic resonance	[30]
Co-precipitation	20 nm cubic and spherical nanoparticles	high	Hc decreases to zero	20–150 °C	Biomedical application	[20]
Co-precipitation	8–25	27	32.09	40 °C	Catalyst	[43]
Co-precipitation	15 approximately spherical shape	70	5	Heated, 80 °C	Biomedical application	[44]
Co-precipitation	almost spherical with diameters ranging from 10 to 30 nm.	65.53	189.75, 126.92 136.97 0	80 °C	Hyperthermia treatment of cancers	[45]
Co-precipitation	5–150 nm spherical and cubic geometry, agglomerated nanoparticles	48–71	15–41	94–98 °C	Contrast agent in medical imaging	Our work

## Data Availability

All data analyzed during this study are included in this published article.
